# Progress in Surgery

**Published:** 1898-05-28

**Authors:** 


### Progress in Surgery.

### GENERAL SURGERY.

(*Continued from page* 116).

**Antiseptic Precautions.**—During the past five months Dr. Landerer and Dr. Krämer[7] have used for disinfection of the field of operation a 1 per cent. solution of formalin with very good results. Compresses dipped in this solution and covered by some impermeable material are applied to the skin and allowed to remain with one or two renewals from twelve to thirty-six hours. The micro-organisms existing in the cutaneous glands are thereby attacked. This use of formalin is only a preliminary measure, the careful cleansing and disinfection of the skin immediately before the operation should not be omitted. Dr. W. W. Keen[8] has employed with very satisfactory results the cotton gloves for surgical operations, seeing that the hands cannot be rendered perfectly aseptic. They were originally recommended by Professor Mikulicz, of Breslau, and described in The Hospital of December 18th, 1897, p. 206.

**Fractures and Dislocations.**—After reviewing the various methods for the treatment of fractures, G. V. Bergmann[9] summarises the following principles: (1) True adaptation by extension, counter extension, and careful manipulation; (2) immediate attention to exudation of blood, especially about joints, by careful massage; (3) fixation of fragments by splints of various forms that are cheapest, and most readily placed in position with the certainty of producing good results; (4) fixation of the joints on either side; (5) extension apparatus is necessary in oblique fractures with marked overlapping of fragments and shortening of limb; (6) commencing atrophy must be counteracted by changes of splints and early massage; (7) massage and passive motion must be commenced as soon as possible in fractures involving joints on account of the danger of loss of function; and (8) in compound fractures the greatest care should be given to the surroundings; the hair should be shaved and aseptic conditions surround the entire areas. According to G. A. Wright,[10] the whole treatment of fractures needs revision—not on the lines of wiring or pegging or screwing simple fractures, unless in exceptional cases, but as to the duration of treatment by splints. If the time be largely reduced during which fractures are kept up and active movements begun within the limits of pain, carefully avoiding all violent passive movements, better and quicker results will be obtained without resort to more dangerous methods. Forcible passive movement is fraught with danger. Its ill effects are seen in their grossest form in the work of bone setters; but it would be well for surgeons to consider what happens when they attempt to restore the mobility of a joint which is stiff from the result of a recent fracture, involving it or its neighbourhood, by forcible passive movement. In such a proceeding young tissue of repair is lacerated, and each time a fresh subcutaneous wound is inflicted. Every such wound means the formation of cicatricial tissue, which, when it heals, is apt to bind together still more closely the structures injured, and too often the final result is greater rigidity than at first. Pain, the index of injury, points to the unwisdom of the practice. On the other hand, active movement within the limits of pain gently stretches the young tissue, and does not reopen the wound. These remarks in no way refer to old injuries with localised bands of adhesion which require severance to set the part free. The use of massage, advocated so strongly in France by M. Lucas-Championnière in the treatment of recent fractures, has not received the general attention in this country which, in W. H. Bennett's[11] opinion, it deserves. Massage, in any ordinary case, if properly applied, can be used without producing any movement between the bone ends worth mentioning, and in the most difficult cases the amount of movement is not sufficient to delay union, for union, he believes, occurs, *cæteris paribus*, more rapidly in cases treated by massage than in those treated by conventional plans. Indeed, he says, it is permissible to raise the question whether, under any circumstance, slight movement between the fragments, provided that the position of the parts is good, is not rather conducive to union than the reverse, when it is remembered that in many fractures in which union is slow consolidation rapidly takes place when some mobility near the bone ends is brought about, either by encouraging the active use of the limb or by passive movements. The stiffness and pain which follow in many of these cases is often erroneously attributed to adhesion in or about the joint, or to some slight faulty position of the fractured bone. In reality the defective movement is, he believes, practically always due to matting of the soft parts immediately about the line of fracture. In the treatment by massage this matting is impossible; the tendons are prevented from becoming adherent, the muscles do not waste, the joints are kept supple, and nerves cannot become implicated in adhesions. This condition of things, when compared with the state of the limb upon the removal of the splints in a case treated by the usual method of immobility, is sufficient to entitle massage to a fair claim as a routine treatment in a large number of recent fractures. Mr. Bennett quotes several cases in which this method of treatment was adopted with good results. The technique of the treatment has been fully described over three years ago in The Hospital. The *Lancet*[12] very correctly states that, unless the structure and outline of the bone are perfectly restored, its function is more or less impaired, and we view with suspicion any method of treatment which in any way seems to lessen the importance of this primary consideration. Given perfect correction of displacement, it is usually an easy matter to maintain it, and then it cannot be doubted that massage practised even quite early will be useful. But if massage is exalted into the chief place in treatment, and correction of displacement is regarded as of only minor importance, its wider employment will be attended with much harm. In a concise account of the surgical application of the X rays J. W. White[13] states, as regards the bones of the extremities, that fractures and dislocations (with some exceptions) can be readily demonstrated. Skiagraphy is of great practical importance in fractures attended with so much swelling of surrounding tissues that satisfactory palpation of the fragments is impossible, in fractures about joints where crepitus is so often absent and preternatural mobility readily confused with joint motion; in epiphysial separations, and in a few special fractures, *e.g.*, fracture of the femoral neck. In the disability following old fractures near joints and complicated laxations he has found skiagraphs to be of the greatest help in determining the mechanical cause of the limitation of motion and in deciding whether excision of the joint involved or some less radical procedure should be adopted. He unhesitatingly answers in the negative the question whether or not the patient has the right to demand, as ordinary care, that the medical attendant should have a skiagraph of the fracture taken. Until a much larger number of cases have been observed, and the pictures and the clinical results have been compared, the routine use of skiagraphy might be more harmful than useful. C. T. Dent[14] thinks that impartial consideration of the value of skiagraphy in injuries of the bones and joints seems to show that its use is more limited than at first appeared probable. His own experience is that the rays are of comparatively little use, save for rather coarse or marked lesions. As an aid to the precise diagnosis and treatment of many ordinary fractures, such as are easily examined by the rays, skiagraphy has as yet scarcely been sufficiently employed, though its value would seem obvious enough. Here the use of the screen is invaluable. A certain amount of longitudinal splitting in addition to the common oblique fracture of the bone is far more often the case than is imagined; in greenstick fracture this splitting may be always observed. The splitting may merely amount to a fissure of the shaft, and need not be complete, but examination of fractures by means of the rays will show that very frequently indeed a longitudinal fragment of bone is detached. In fact, a skiagraph will constantly prove that a fracture supposed to be transverse and simple is really oblique and comminuted. J. B. Roberts[15] remarks that since one of the effects of circulating changes in osseous tissue is a tendency to encourage growth of bone, this physiological fact seemed to him to present a possible means of increasing the formation of callus at the seat of fracture. In a case of delayed union of the tibia or fibula he carried out this suggestion with some measure of success by keeping the limb in a state of chronic congestion, by having the patient wear a rubber bandage round the lower part of the thigh, applied so tightly that the foot and leg would be kept blue and swollen from congestion of the veins. This bandage was worn for nearly three and a half months. The result of experimental investigation upon animals regarding the influence of neurectomy upon callus formation is recorded by Kapsammer.[16] He concluded that after resection of the sciatic nerve fractures of the leg are healed with abnormally great callus formation, but that the consolidation of this callus occurs later than with normal innervation. Kusmin, who had previously investigated the same point, came to the conclusion that the callus after the neurectomy was both more abundant and of greater firmness.

[7] Epit. Brit. Med. Jour., March 19, 1898, and Centralbl für Chir., No. 8, 1898. [8] Annals of Surgery, Feb., 1898, p. 224. [9] Amer. Jour. Med. Sci., Dec., 1897, and St. Petersburger Med. Woch., 1897, No. 14. [10] Med. Chron., Feb. 1898, p. 330. [11] Lancet, Feb. 5, 1898, p. 359. [12] Ibid., p. 381. [13] Amer. Jour. Med. Sci 1898, p. 6. [14] Practitioner, Feb., 1898, p. 123. [15] Annals of Surgery, Jan., 1898, p. 95. [16] Wien Klin. Woch., 1897, No. 31; and Epit. Brit. Med Jour., Feb. 1898, p. 30.

